# MicroRNA-21 promotes head and neck squamous cell carcinoma (HNSCC) induced transition of bone marrow mesenchymal stem cells to cancer-associated fibroblasts

**DOI:** 10.1186/s12885-023-11630-7

**Published:** 2023-11-22

**Authors:** Hao Wang, Zhengyu Zhou, Wenchao Lin, Yechun Qian, Shifang He, Jun Wang

**Affiliations:** 1 Department of Otorhinolaryngology, Ruijin Hospital Lu Wan Branch, Shanghai Jiao Tong University School of Medicine, Shanghai, 200020 China; 2 Department of Laboratory Diagnostics, Changhai Hospital, Naval Medical University, Shanghai, 200433 China; 3 Department of Otolaryngology & Head and Neck Surgery, Ruijin Hospital, Shanghai Jiao Tong University School of Medicine, Shanghai, 200020 China

**Keywords:** MicroRNA-21, Head and neck squamous cell carcinoma, Human bone marrow mesenchymal stem cells, Exosomes, Cancer-associated fibroblasts

## Abstract

**Background:**

Most patients diagnosed with head and neck tumor will present with locally advanced disease, requiring multimodality therapy. Bone marrow-derived mesenchymal stromal cells (BMSCs) respond to a variety of tumor cell-derived signals, such as inflammatory cytokines and growth factors. As a result, the inflammatory tumor microenvironment may lead to the recruitment of BMSCs. Whether BMSCs in the tumor environment are more likely to promote tumor growth or tumor suppression is still controversial. We aimed to determine whether microRNA-21(miR-21) would play a vital role in HNSCC induced transition of human bone marrow mesenchymal stem cells (hBMSCs) to cancer-associated fibroblasts (CAFs).

**Methods:**

In this study, we used electron microscope to observed exosomes collected from human tissue and two cell lines. We co-cultured hBMSCs with exosomes from FaDu and Cal-27 cells with miR-21 inhibited or not, then assessed cell cycle changes of hBMSCs with flow cytometry and determined expression level of α-SMA and FAP through qRT-PCR and Western blot.

**Results:**

We observed an up-regulation of miR-21 expression in HNSCC tissue and FaDu and Cal-27 cells. Importantly, the exosomes derived from both cells induced CAFs-like characteristics in hBMSCs. while treatment with a miR-21 inhibitor effectively suppressed the transition of hBMSCs to CAFs and reversed the changes in the cell cycle distribution. This suggests that miR-21 plays a crucial role in facilitating the transition of hBMSCs to CAFs and modulating the cell cycle dynamics.

**Conclusion:**

Our findings highlight the significance of miR-21 in mediating the communication between HNSCC cells and hBMSCs through exosomes, leading to the promotion of CAFs-like features and alterations in the cell cycle of hBMSCs.

**Supplementary Information:**

The online version contains supplementary material available at 10.1186/s12885-023-11630-7.

## Introduction

Head and neck squamous cell carcinoma is relatively rare and has become one of the tumors with the worst prognosis in the head and neck cancer [1]. It has been reported that the 5-year survival rate of Head and neck squamous cell carcinoma patient is 30–35% approximately [2]. Given the challenges posed by current treatment methods, there is a need to explore new and effective therapies.

In recent times, hBMSCs have gained attention as targeted vectors for gene therapy in HNSCC due to their inherent homing, trafficking potential, and regenerative capacity [3, 4]. However, the role of hBMSCs in tumor development, specifically their homing into tumor tissue, remains controversial. Some studies suggest that hBMSCs transferring into the cancer microenvironment can be recruited and activated into cancer-associated fibroblasts, which may contribute to cancer formation and development [5]. These findings raise concerns about the potential risks associated with using hBMSCs as targeted vectors in this context. miRNAs play a crucial role in mediating cancer development and proliferation. In fact, Taylor et al. were the first to propose the diagnosis of ovarian cancer through the detection of miRNA in blood exocrines [6]. It has been discovered that miRNAs derived from tumor exosomes facilitate the exchange of information between tumor cells and the microenvironment of metastatic targets. Additionally, miRNAs serve as significant regulators of cancer cell proliferation and apoptosis [7–9], while also playing a vital role in the processes of cancer cell metastasis, development, and invasion [10–12].

MiRNA-21 is widely recognized as a carcinogenic small RNA [13]. Literature has reported an abnormal increase in miRNA-21 expression levels in various cancer specimens, including hepatocellular cancer [14], breast cancer, gastric cancer and lung cancer [15–17]. Additionally, it has been observed that exosomal miR-21 from hepatocellular cancer can stimulate the transition of hepatocyte stellate cells to CAFs [18]. Hence, our objective is to investigate whether miR-21 also plays a role in the transition of hBMSCs to CAFs. Studies have demonstrated that miRNA-21 promotes cancer development by regulating tumor-related genes, such as SATB1, S100A9 [19].

In our study, we evaluated the expression level of miR-21 in human cancer tissue and two cell lines. Through co-culturing hBMSCs with exosomes derived from FaDu and Cal-27 cells, we discovered that miR-21 was capable of promoting the transition of hBMSCs into CAFs. Furthermore, we identified that miR-21 could regulate the cell cycle dynamics of hBMSCs. These results suggested that miR-21 is necessary for the HNSCC induced transition of hBMSCs to CAFs.

## Materials and method

### Tissue samples

HNSCCspecimens were obtained from cancer patients, while normal tissue specimens were obtained from paracancerous tissues. Informed consent forms were completed by all patients. Samples were promptly frozen at -80 °C upon collection and were only thawed 10 min before any biochemical analysis was conducted. This study involving human participants underwent review and approval by the Ruijin Hospital Luwan Branch Ethics Committee, Shanghai Jiao Tong University School of Medicine, on June 11, 2020. All patients provided written informed consent and agreed to participate in our study.

### Cell culture

FaDu, Cal-27 cells and 293 T cells were obtained from the American Type Culture Collection (ATCC, Manassas, VA, USA) and maintained in Eagle's Minimum Essential Medium (Gibco, Grand Island, NY, USA) supplemented with 15% fetal bovine serum. The cells were incubated at 37 °C in a carbon dioxide (CO_2_) incubator with a 5% CO_2_ atmosphere. hBMSCs were cultured in complete mesenchymal stem cell culture medium (Gibco, Grand Island, NY, USA) containing 10% fetal bovine serum and 1% penicillin–streptomycin at 37 °C in a 5% CO_2_ incubator.

### Isolation and identification of exosomes

Tumor tissue was gently sliced into small fragments (1–2 mm) and incubated for 30 min at 37 °C either in plain RPMI-1640 media supplemented with collagenase D (2 mg/ml, Roche) and DNase I (40 U/ml, Roche). After a filtration step (70 μm), cells and tissue debris were further eliminated by centrifugation at 300 × g for 10 min and 2000 × g for 20 min. Supernatants were centrifuged at 16,500 × g for 20 min and 118,000 × g for 2.5 h to collect large and small exosomes, respectively (Type45 Ti rotor, k-factor 1279.1 and 178.6, respectively, Beckman Colter). All centrifugations were performed at 4 °C. The large exosomes and small exosomes enriched pellets were resuspended in PBS for further analysis. For exosomes isolation from culture system, supernatant collected from 3-day cell cultures was first centrifuged at 500 × g for 10 min to remove any cell contamination. Next, the upper supernatant was further centrifuged at 12,000 × g for 20 min to remove any possible apoptotic bodies and large cell debris. Final centrifugation was performed at 100,000 × g for 70 min to enrich exosomes, and the pellet was rinsed in 20 mL of PBS. Finally, exosomes were collected by ultracentrifugation at 100,000 × g for 70 min. The number and morphology (cup-shaped) of exosomes were examined using a NanoSight NS300 microscope (Malvern Instruments Ltd., UK) and a Philips CM120 BioTwin transmission electron microscope (FEI Company, USA), respectively. For concentration detection, exosomes were lysed with lysis buffer (50 mM Tris–HCl, pH 7.4, 150 mM NaCl, 1% NP-40, and 0.1% sodium dodecyl sulfate) supplemented with protease inhibitor cocktail set I (Biotool, Jupiter, FL, USA) and phenylmethanesulfonyl fluoride (PMSF, Sigma-Aldrich). Total protein contents of the lysates were measured using the BCA protein assay kit (Pierce, Rockford, IL, USA).

### Quantitative real-time PCR

Total RNA was prepared from the specimens with a TriZol reagent (Invitrogen, California, USA). For the analysis of α-SMA, FAP and GAPDH, the reaction was performed using a real-time PCR System (Applied Biosystems, Foster City, CA, USA). Primer sequences are presented as follows:

- α-SMA (Forward primers 5'-ACT GCC TTG GTG TGT GAC AA-3'; Reverse primers 5'-TCC CAG TTG GTG ATG ATG CC-3')
- FAP (Forward primers 5'-AGA ACC ATG CTT TGG AGA TAC T-3'; Reverse primers 5'-GGT GGA TCT CCT GGT CTT TGT-3')
- GAPDH (Forward primers 5'-AAT CCC ATC ACC ATC TTC-3'; Reverse primers 5'-AGG CTG TTG TCA TAC TTC-3')

### Flow cytometry

Cell samples were collected and fixed, followed by the addition of 500μL staining buffer, 10uL RNase A, and 25 μL propidium iodide solution. The samples were then incubated in the dark at 37 °C for 30 min. Flow cytometry analysis was performed within 24 h after staining. Red fluorescence emitted by the samples was detected using a flow cytometer (Beckman, Brea, CA, USA) at an excitation wavelength of 568 nm, corresponding to the FL2 detection channel of the flow cytometer. Light scattering was also simultaneously measured. Subsequently, cell cycle analysis was conducted using FLOWJO software.

### Western blotting

Briefly, hBMSCs were treated with lysis buffer containing 10 mM HEPES, pH 7.4, 2 mM EGTA, 0.5% NP-40, and protease inhibitors. A total of 20 μg of total proteins were loaded onto SDS-PAGE gels, followed by transfer onto nitrocellulose membranes. The presence of proteins was detected using specific primary antibodies against α-SMA, FAP, and β-actin (Sigma-Aldrich, St. Louis, MO, USA). Subsequently, corresponding HRP-conjugated secondary antibodies (Servicebio, Wuhan, China) were applied, and visualization was performed.

### Statistical analysis

We used GraphPad Prism 5 to perform the statistical analysis and displayed the data as the means ± SD. We used student's t test and two-way ANOVA to calculate the statistical significance. We consider the value of *P* < 0.05 as statistically significant (*P < 0.05; **P < 0.01; ***P < 0.001; ****P < 0.0001).

## Results

### miR-21 was up-regulated in hypopharyngeal cancer tissue and tumor cell lines

**Fig. 1:**
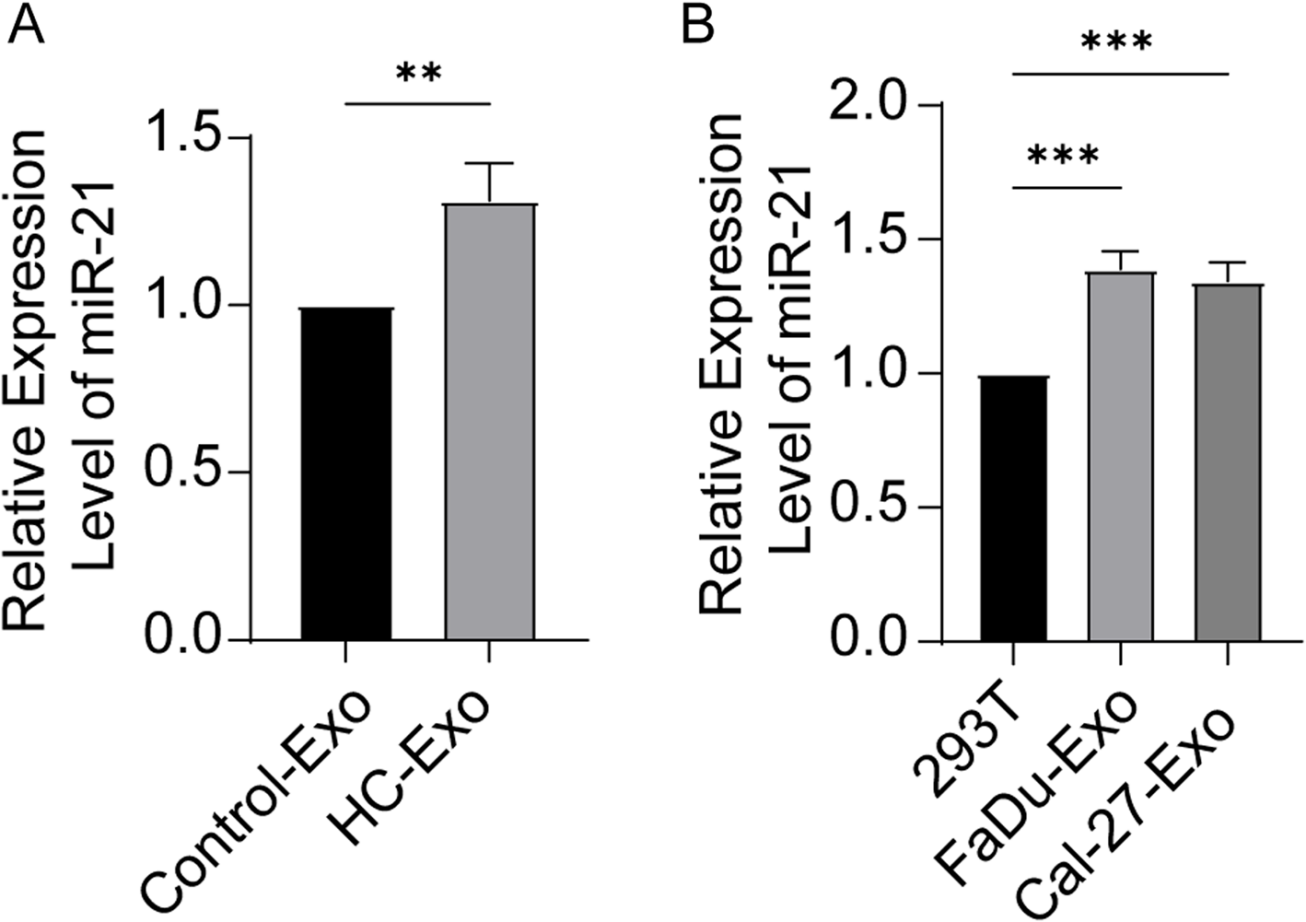
Up-regulation of miR-21 in HNSCC and cell lines. **A** The expression level of miR-21 in HNSCC and control tissue analyzed by qRT-PCR (Unpaired t test, *n* = 53, α = 0.05, *P* = 0.0025). **B** The expression level of miR-21 in FaDu, Cal-27 cells and control (293 T cell) analyzed by qRT-PCR (Unpaired t test, *n* = 3, α = 0.05, *P* = 0.0015)

We assessed the expression levels of miR-21 in tissue samples from 53 patients with HNSCC, using paracancerous tissue samples as a control. Additionally, we compared the expression levels of miR-21 in theHNSCC cell line FaDu and Cal-27 cells with those in a normal cell line (293 T cells) as a control. Quantitative reverse transcription-polymerase chain reaction (qRT-PCR) analysis revealed a significant increase in miR-21 expression levels in both HNSCC tissue samples (Fig. 1A) and FaDu, Cal-27 cells compared to their respective controls (Fig. 1B).

### Isolation and identification of exosomes from hypopharyngeal cancer

**Fig. 2:**
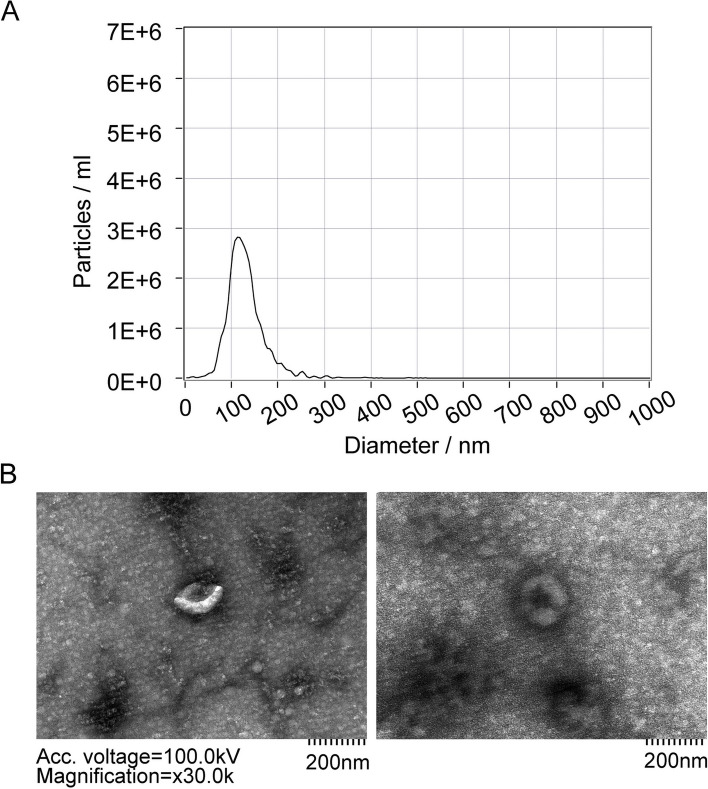
Isolation and identification of exosomes from HNSCC (**A**) Particle size distribution curve of diluted exosomes (*n* = 10). **B** Exosomes observed by electron microscope

Exosomes derived from HNSCC tissues were collected and subsequently diluted 1000-fold. The concentration and diameter of these exosomes were analyzed, and electron microscopy was used to capture images (Fig. 2A and B). The particle size distribution curve and Table 1 revealed that the majority (97.8%) of the diluted exosomes had a diameter of 117.1 nm, with a corresponding concentration of 2.8E + 06 particles/ml.

**Table 1:** Particle size distribution of exosomes from HNSCC

Diameter/nm	Particles/mL	FWHM/nm	Percentage
117.1	2.80E + 06	59.7	97.8
251	1.20E + 05	14.9	1.1
306.2	4.20E + 04	13.9	0.4
13.5	2.20E + 04	10	0.1
328.7	1.60E + 04	12.1	0.2

### Exosomes from FaDu, Cal-27 cells mediated induction of CAF-like features in hBMSCs

**Fig. 3:**
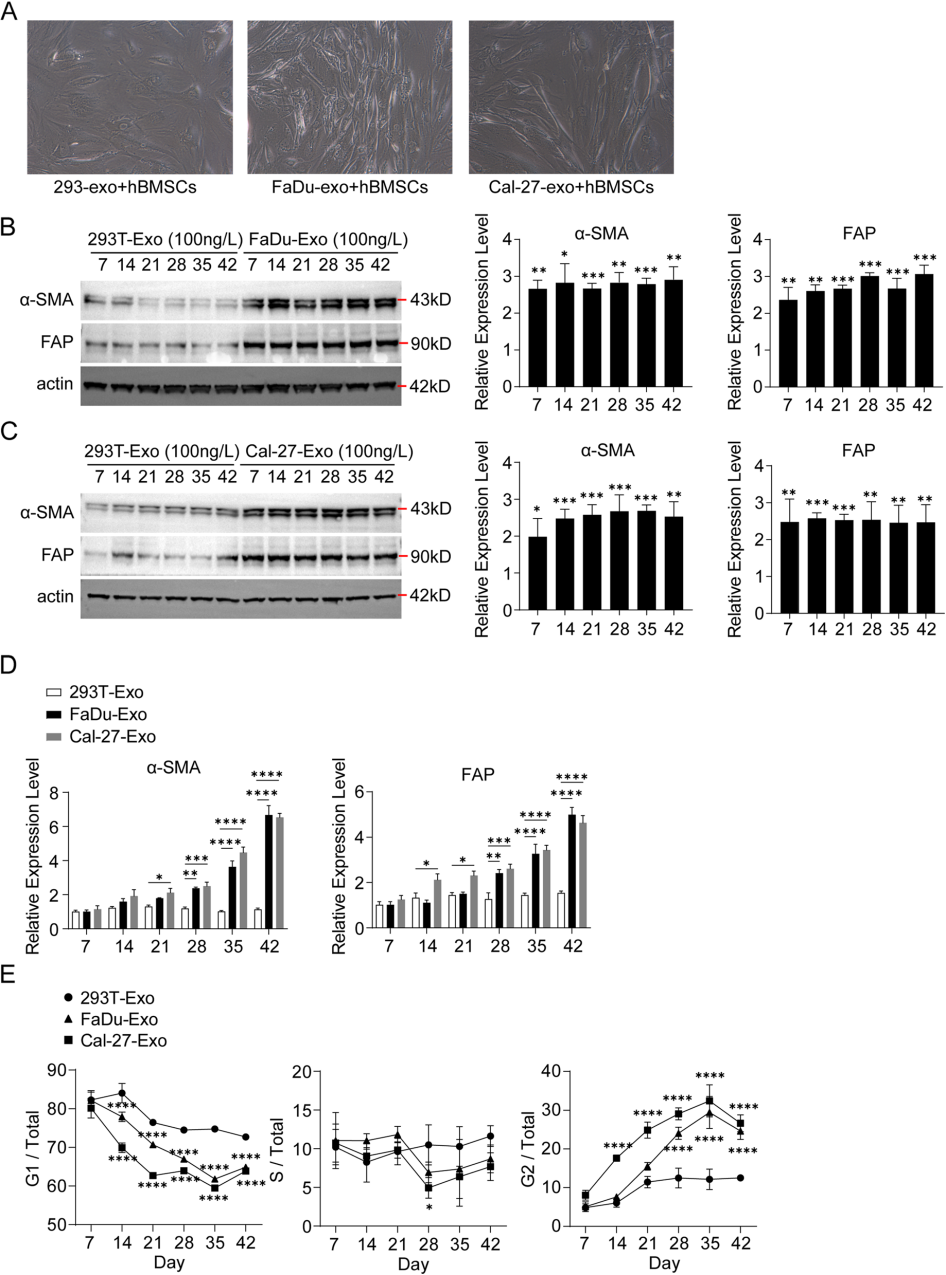
Exosomes from FaDu and Cal-27 cells promoted hBMSCs’ CAFs-like features. **A** Growth state of hBMSCs observed by microscope. **B** Expression level of α-SMA and FAP in hBMSCs on different days analyzed by Western blot. **C** The α-SMA and FAP expression level on different days in hBMSCs analyzed by qRT-PCR (Two way ANOVA, *n* = 3, α = 0.05, **P < 0.01, ****P < 0.0001). **D** Cell proportion of G1, S and G2 phase analyzed by flow cytometry (Two way ANOVA, *n* = 3, α = 0.05, *P < 0.05, ***P < 0.001, ****P < 0.0001

To investigate the impact of exosomes derived from FaDu and Cal-27 cells on the transition of hBMSCs to CAFs, we collected exosomes from both FaDu, Cal-27 cell, and 293 T cell culture systems. Subsequently, hBMSCs were co-cultured with the collected exosomes, and the expression of CAF-related markers was evaluated at various time points (day 7, 14, 21, 28, 35, and 42). Microscopic observations revealed a significant increase in the number of CAF-like cells in the FaDu and Cal-27 derived-Exo + hBMSCs group compared to the control (Fig. 3A). The morphology of hBMSCs exhibited a tendency to adopt a spindle or star-shaped flat structure, and they differentiated into fibroblasts with evident protein synthesis and secretion activities. Western blot results demonstrated a significant upregulation of CAF-related proteins, including α-SMA and FAP, in hBMSCs of the FaDu-Exo + hBMSCs group (Fig. 3B) and Cal-27-Exo + hBMSCs (Fig. 3C) compared to the control. qRT-PCR results confirmed an increased expression of miR-21 in hBMSCs of the FaDu and Cal-27 derived-Exo + hBMSCs group compared to the control (Fig. 3D). Flow cytometry results indicated a significant decrease in the proportion of cells in the G1 phase and a notable increase in the proportion of cells in the G2 phase in the FaDu and Cal-27 derived-Exo + hBMSCs group compared to the control (Fig. 3E).

### Exosomes from FaDu and Cal-27 cells treated with miR-21 inhibitor reversed induction of CAF-like features in hBMSCs

**Fig. 4:**
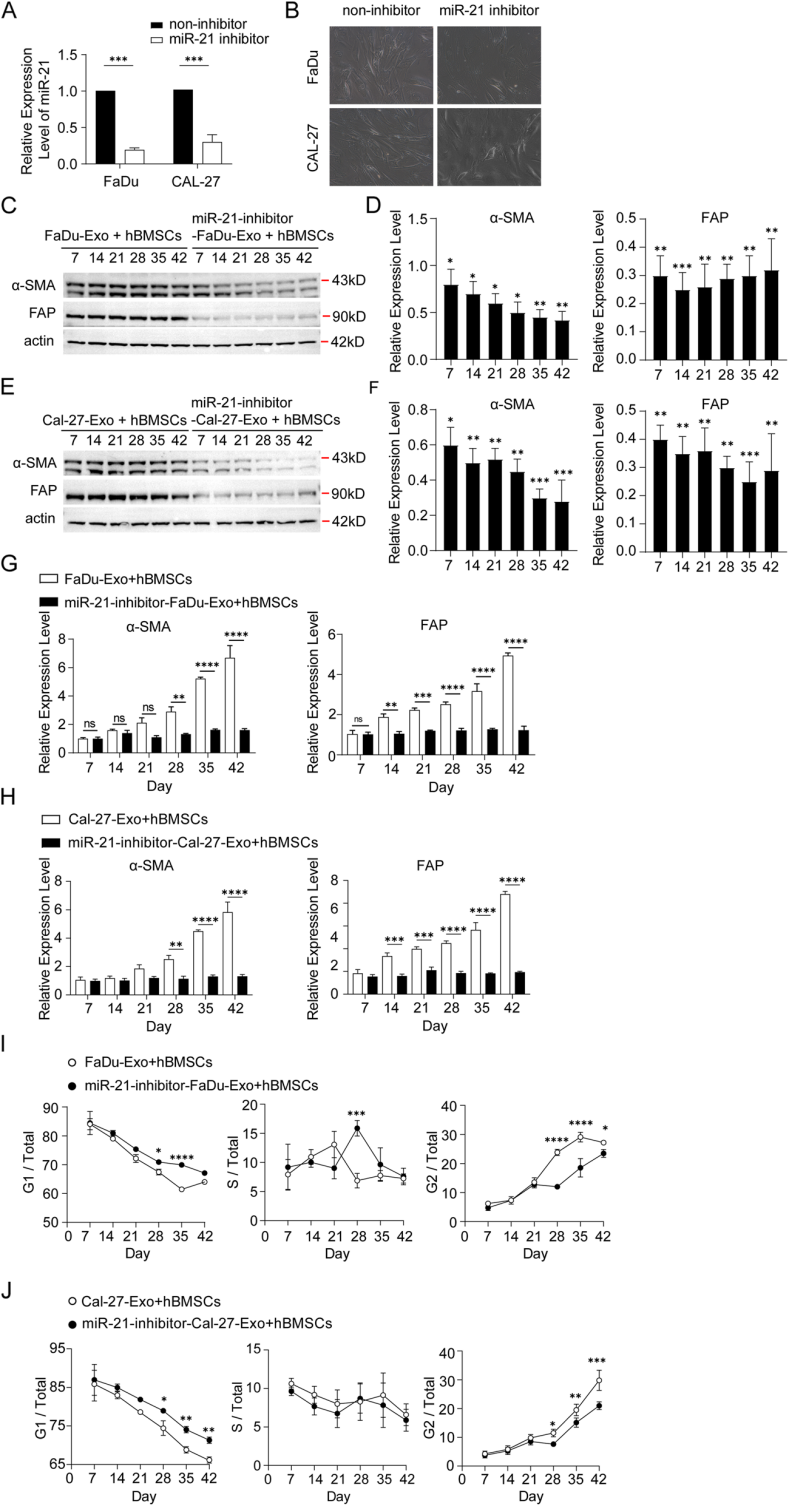
Exosomes with knock-out of miR-21 inhibited CAFs-like features in hBMSCs. **A** The expression level of miR-21 in exosomes from FaDu and Cal-27 cells treated with miR-21 inhibitor or not were analyzed by qRT-PCR (Unpaired t test, *n* = 10, α = 0.05, *P* = 0.0008). **B** Growth state of hBMSCs observed by microscope under treatment with miR-21 inhibited exosomes from FaDu and Cal-27 cells. **C**-**F** The α-SMA, FAP and β-actin expression level of hBMSCs analyzed by Western blot and qRT-PCR (G-H) on different days (Two way ANOVA, *n* = 3, α = 0.05, **P < 0.01, ***P < 0.001, ****P < 0.0001). (I-J) Cell proportion of G1, S and G2 phase analyzed by flow cytometry (Two way ANOVA, *n* = 3, α = 0.05, *P < 0.05, **P < 0.01, ***P < 0.001, ****P < 0.0001

To investigate the role of miR-21 in the transition of hBMSCs to CAFs, we collected exosomes from the FaDu and Cal-27 cell culture system, which were treated with miR-21 inhibitor resulting in miR-21 gene knock-out. Subsequently, hBMSCs were co-cultured with the collected exosomes. the expression of CAF-related markers was evaluated at specific time points (day 7, 14, 21, 28, 35, and 42). As revealed in Fig. 4A, qRT-PCR results confirmed a downregulation of miR-21 levels in the collected exosomes from FaDu and Cal-27 cells with miR-21 inhibition. Microscopic observations revealed a significant downregulate in the number of CAF-like cells in the miR-21 inhibited FaDu and Cal-27 derived-Exo + hBMSCs group compared to the control (Fig. 4B). Western blot results indicated a significant decrease in both CAF-related proteins within hBMSCs in the miR-21 inhibitor-treated group compared to the control (Fig. 4C-F). Furthermore, qRT-PCR results confirmed a downregulation of α-SMA and FAP mRNA levels within hBMSCs in the miR-21 inhibitor-treated group compared to the control (Fig. 4G, H). This finding suggests a limited transition from hBMSCs to CAFs, which is consistent with microscopic observations (Fig. 4B). Flow cytometry results demonstrated an increased proportion of cells in the G1 phase and a significantly decreased proportion of cells in the G2 phase in the miR-21 inhibitor-treated group compared to the control (Fig. 4I, J).

## Discussion

Currently, due to their ability to migrate to cancerous tissues, hBMSCs have emerged as promising vectors for targeted gene therapy in various types of cancers [20–24]. Knoop et al. utilized human mesenchymal stem cells as delivery vectors for the sodium iodide symporter in the treatment of hepatocellular cancer, resulting in a 55% reduction in cancer cell survival [20]. Sonabend et al. demonstrated that human mesenchymal stem cells could migrate and deliver targeted molecules to glioma cells [21]. Furthermore, Zhang et al. were the first to discover that hBMSCs could migrate to hypopharyngeal cancer, suggesting their potential as effective targeted delivery vectors for cancer gene therapy [25].

Although hBMSCs show immense potential in targeted gene therapy, research has indicated that hBMSCs can be recruited to cancerous tissues and differentiate into CAFs, which play a significant role in cancer formation and progression [26].

CAFs are a group of fibroblasts found in the cancer stroma, an essential part of the tumor microenvironment. These CAFs promote cancer development, invasion, and metastasis by suppressing the function of immune cells through the secretion of various cytokines and metabolites [26]. Specific marker proteins, such as FAP and α-SMA, are commonly used to identify CAFs.

Furthermore, studies have demonstrated that hBMSCs can accumulate around cancerous tissues and differentiate into pericytes, secreting cytokines and chemokines that stimulate angiogenesis and cancer growth [27, 28]. In gastric cancer, for instance, hBMSCs can be induced to differentiate into CAFs through the involvement of miR-141. MiR-141 suppresses the development of gastric cancer cells and inhibits the differentiation of hBMSCs into CAFs by targeting STAT4 [29]. Similarly, 4T1 breast cancer cells can enhance the migration of hBMSCs, but this migration can be inhibited by the presence of antibodies neutralizing basic fibroblast growth factor (bFGF) [30].

Our findings, as shown in Fig. 3, demonstrated that co-culture of hBMSCs with exosomes derived from the FaDu and Cal-27 cell line, could induce the differentiation of hBMSCs towards CAFs. The expression levels of FAP and α-SMA, as assessed by Western blot and qRT-PCR, were significantly higher in the FaDu and Cal-27-exo + hBMSCs group compared to the control group. These results indicated that certain exosomes released by FaDu and Cal-27 cells may have the potential to induce the differentiation of hBMSCs into CAFs. Flow cytometry analysis revealed a significant decrease in the proportion of cells in the G1 phase and a significant increase in the proportion of cells in the G2 phase in the FaDu and Cal-27-exo + hBMSCs group compared to the control group (Fig. 3E). This observation suggests that a higher number of hBMSCs tended to differentiate into CAFs in the FaDu and Cal-27-exo + hBMSCs group compared to the control group.

Numerous studies have indicated that the abnormal expression of miRNAs contained in exosomes can serve as potential biomarkers for cancer diagnosis and prognosis [31]. For instance, Taylor et al. conducted a miRNA map analysis and observed a significant difference between the ovarian cancer patient group and the normal control group. The miRNAs isolated from exosomes in the serum of ovarian cancer patients exhibited a strong correlation with the miRNAs extracted from ovarian cancer tissue [6].

The impact of miRNAs on cancer development has been widely studied. They play a crucial role in regulating cancer metastasis by influencing various processes such as cancer cell migration, invasion, angiogenesis, and proliferation [32]. Moreover, miRNAs have been shown to regulate macrophages, an essential component of the innate immune system, affecting cell differentiation, phagocytosis, and apoptosis. This highlights the potential of miRNAs as therapeutic targets in the fight against cancer [33].

Multiple studies have demonstrated that the up-regulation of miR-21 is associated with disease development. Abnormally elevated expression of miR-21 has been detected in various cancer samples and cell lines, including breast cancer, cervical cancer, lung cancer, and pancreatic cancer [34]. As a result, miR-21 is considered an onco-miR [35]. Meng et al. conducted a miRNA microarray analysis and found markedly increased expression of miR-21 in human hepatocellular cancer tissue and cell lines. Inhibiting miR-21 in cultured human hepatocellular cancer cells resulted in increased expression of the PTEN tumor suppressor gene, leading to the suppression of cancer cell proliferation, migration, and invasion [14]. MiR-21 has also been implicated in bladder cancer growth and tumorigenesis, with inhibition of its expression showing promise in suppressing bladder cancer growth [36]. However, the role of miR-21 in HNSCC has not yet been reported. To investigate the potential role of miR-21 in the transition of hBMSCs to CAFs, we utilized a miR-21 inhibitor to suppress the expression of miR-21 in FaDu and Cal-27 cells, from which exosomes were collected and co-cultured with hBMSCs. The results revealed that the expression levels of FAP andα-SMA were significantly lower in the group treated with the miR-21 inhibitor compared to the control group. This suggests that the inhibition of miR-21 restricted the transition of hBMSCs to CAFs, which is consistent with the findings from microscopic observation. It should be noted that in this study, we did not use HNSCC patients-derived exosomes to co-culture with HBMSCs to verify their effect on the fibrotic transformation of hBMSCs. The main reason is that the HNSCC tissues used in this study were removed and placed in the -80°Crefrigerator for a long time, and our adjacent tissues were used as the control group for RNA expression detection. In order to ensure the integrity of the experiment, we did not conduct co-culture experiments. Another reason is that the exosomes from GNSCC patients may be contaminated and cannot be co-cultured with hBMSCS for a long time. Interestingly, flow cytometry analysis showed that the proportion of cells in the G1 phase increased in the miR-21 inhibitor-treated group, while the proportion of cells in the G2 phase significantly decreased compared to the control group. These findings indicate that inhibition of miR-21 may affect the cell cycle progression of hBMSCs during their transition to CAFs.

## Conclusion

In summary, our study provides evidence that miR-21 promotes the transition of hBMSCs to CAFs in HNSCC. additionally, we observed that miR-21 has a significant impact on the cell cycle. These findings contribute to our understanding of the role of miR-21 in tumor microenvironment remodeling and highlight its potential as a therapeutic target in HNSCC.

### Supplementary Information

**Additional file 1.**  

## Data Availability

The data and materials that supports the findings are available on request from the corresponding author.

## Abbreviations

α-SMA: α-Smooth muscle actin
CAFs: Cancer associated fibroblasts
FAP: Fibroblast activation protein
FWHM: Full width at half maximum
hBMSCs: Human bone marrow mesenchymal stem cells
HNSCC: Head and neck squamous cell carcinoma
miR-21: MicroRNA-21
