# Supplementary material for: MicroRNA-21 promotes head and neck squamous cell carcinoma (HNSCC) induced transition of bone marrow mesenchymal stem cells to cancer-associated fibroblasts
Source: BMC Cancer. 2023 Nov 22;23:1135. doi: 10.1186/s12885-023-11630-7 (PMC10666302; doi:10.1186/s12885-023-11630-7)
Supplement: Supplementary file 1 — Additional file 1. [file 12885_2023_11630_MOESM1_ESM.pdf]

Schema

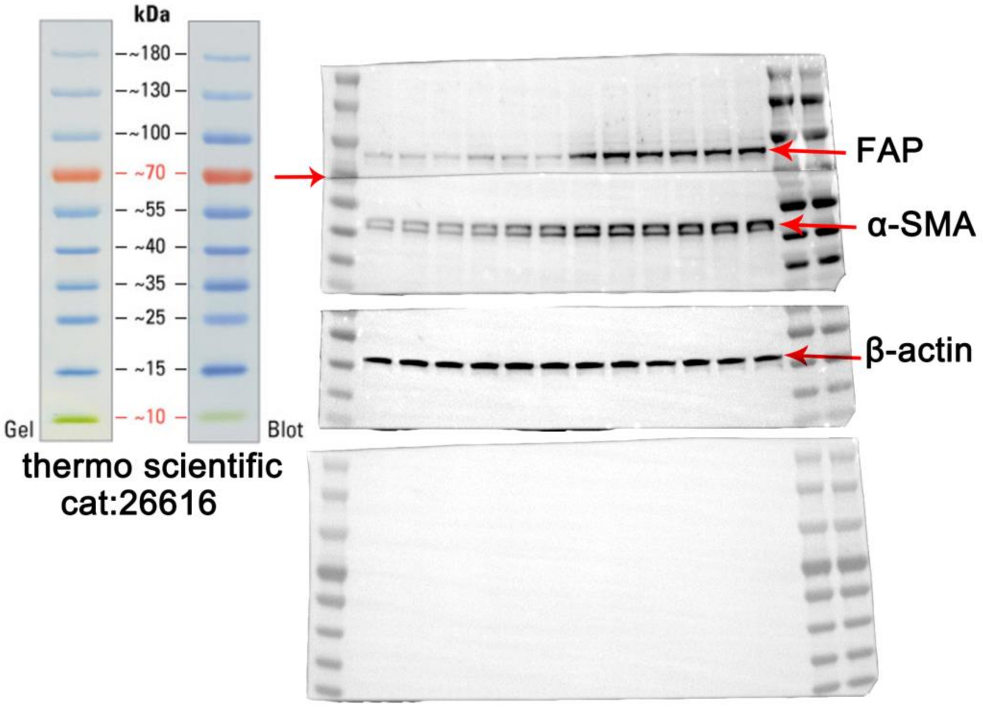

Fig. 3B

1st

FAP	α-SMA
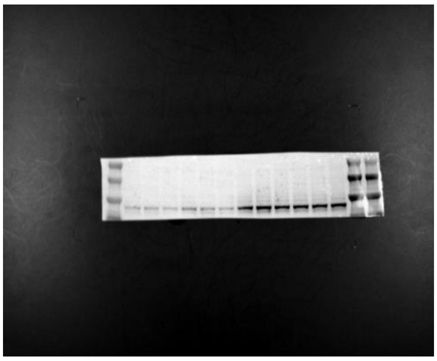	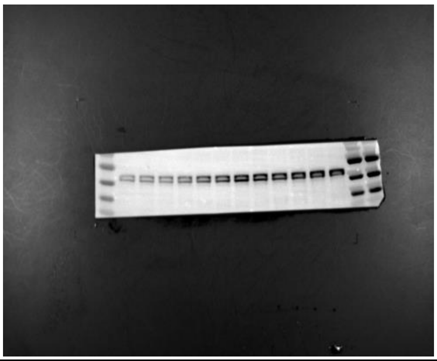
actin	white
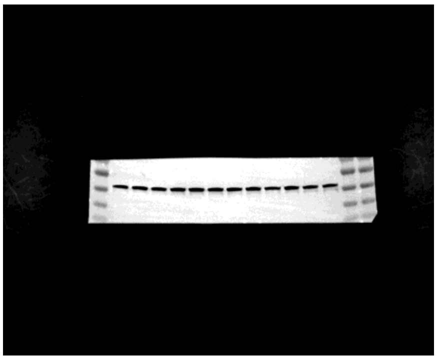	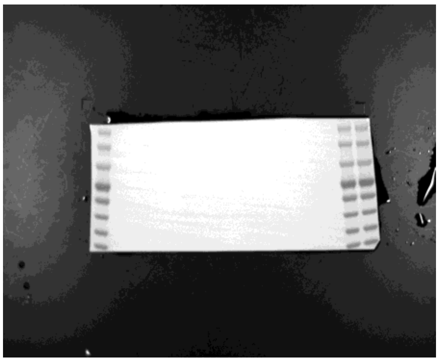

2nd

FAP	α-SMA
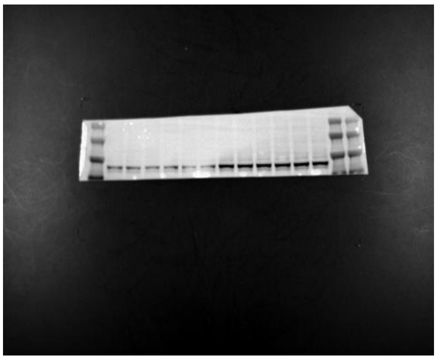	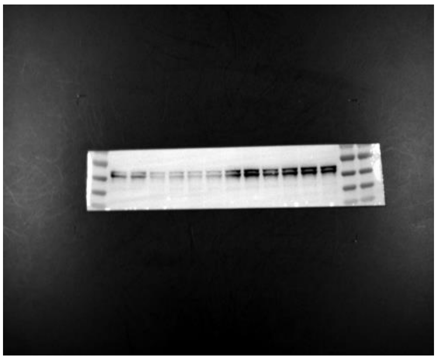
actin	white
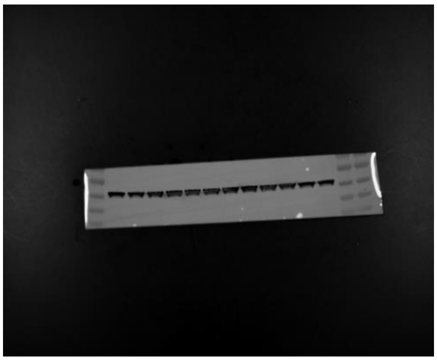	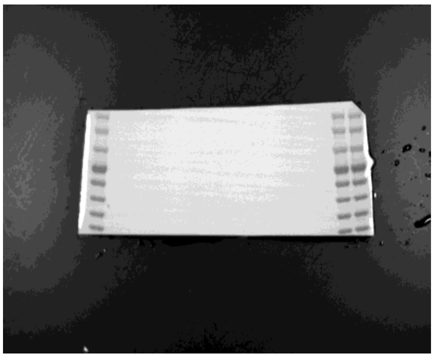

3rd

FAP	α-SMA
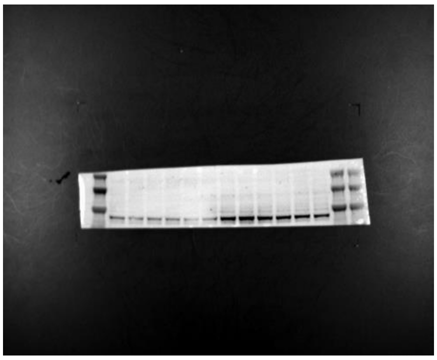	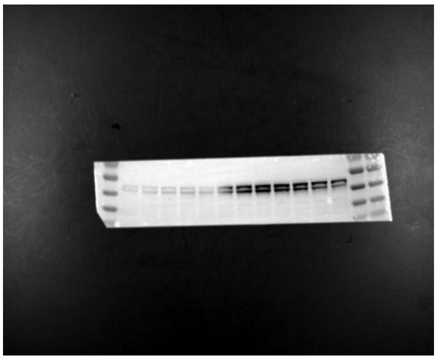
**actin**	**white**
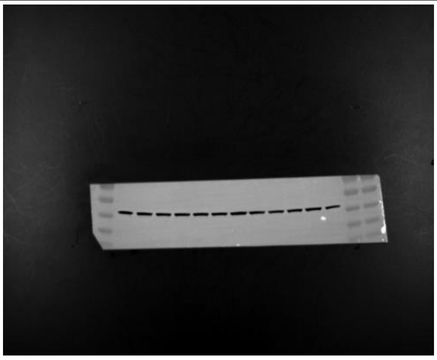	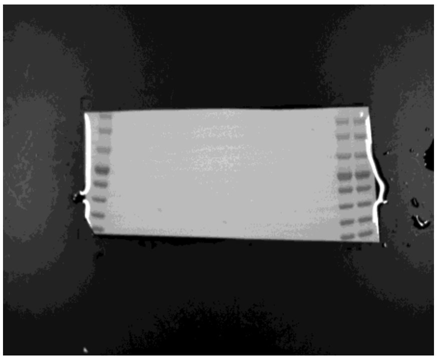

Fig. 3C

1st

FAP	α-SMA
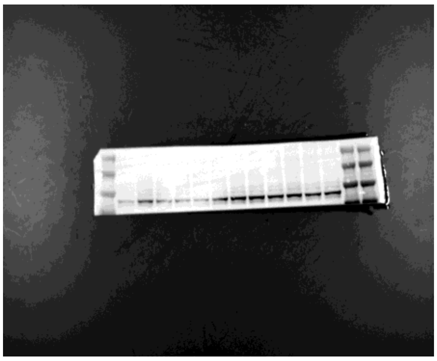	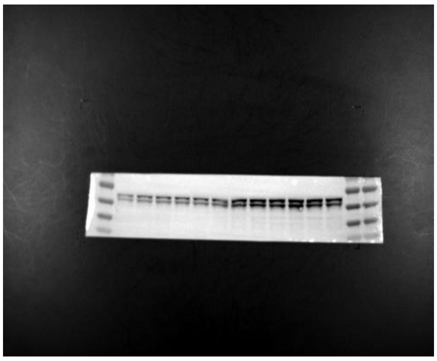
actin	white
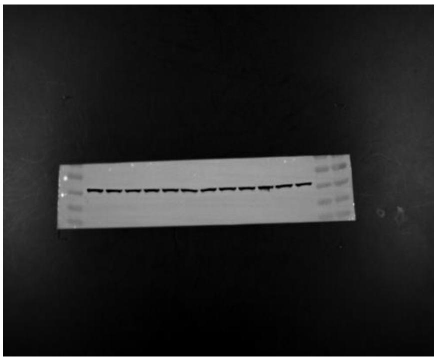	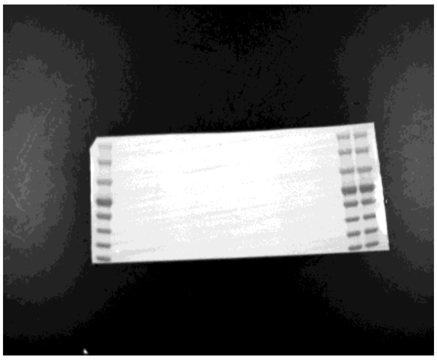

2nd

FAP	α-SMA
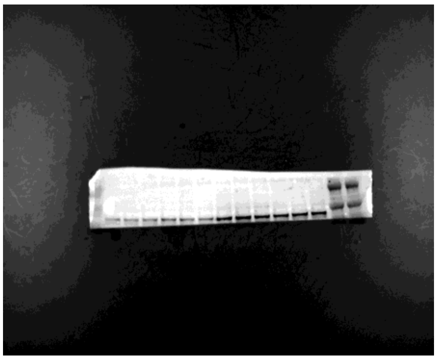	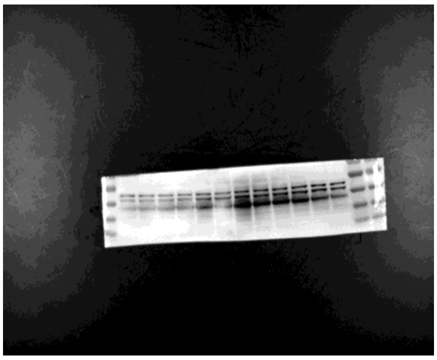
**actin**	**white**
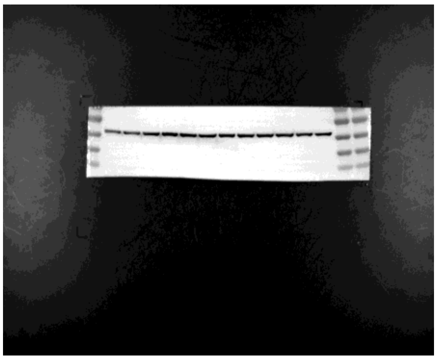	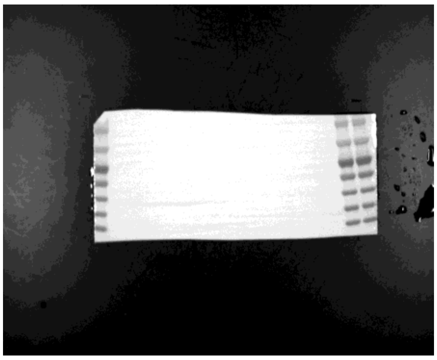

3rd

FAP	α-SMA
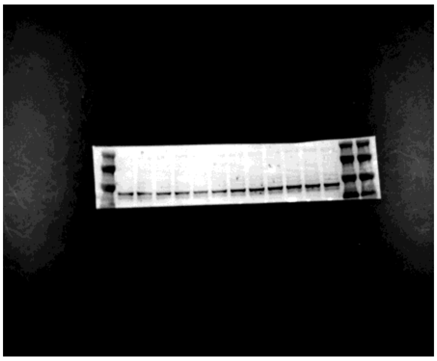	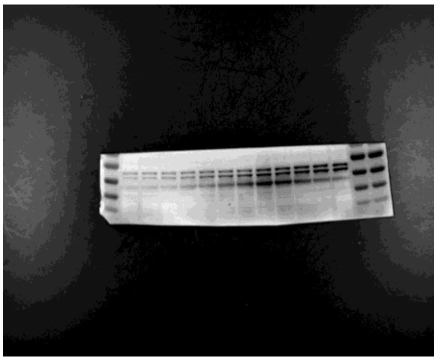
**actin**	**white**
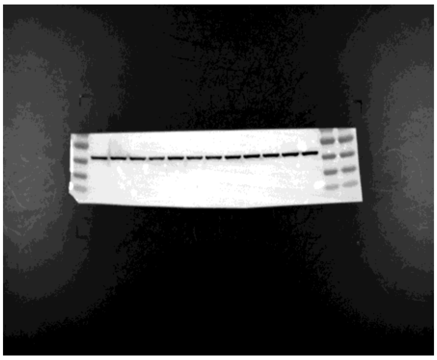	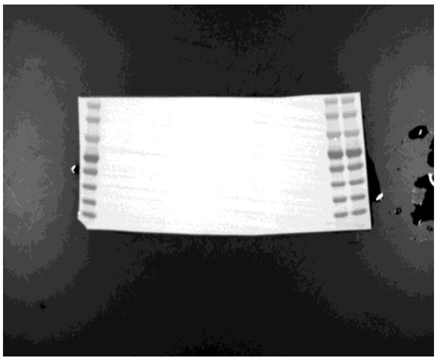

Fig. 4C

1st

FAP	α-SMA
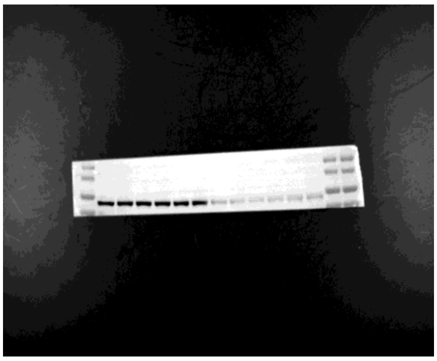	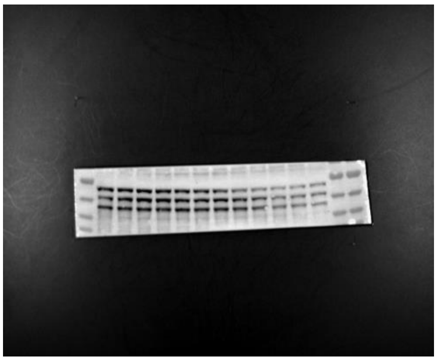
**actin**	**white**
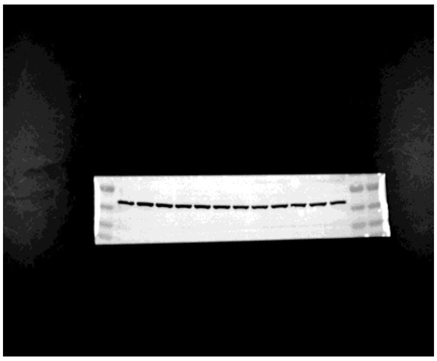	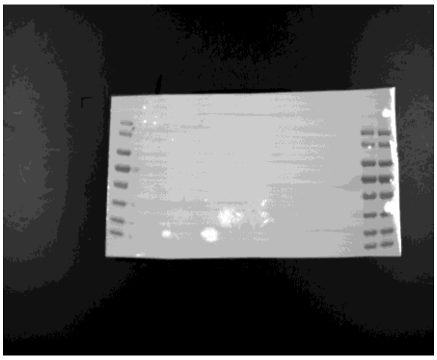

2nd

FAP	α-SMA
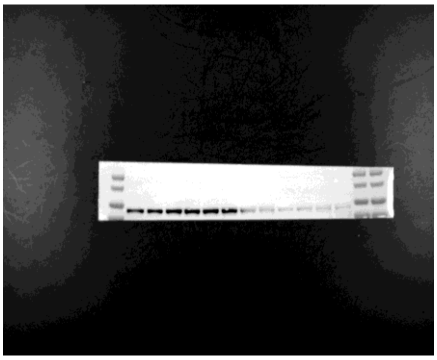	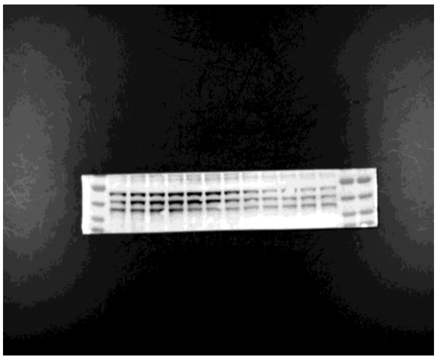
**actin**	**white**
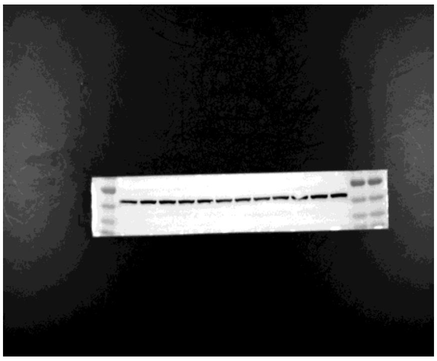	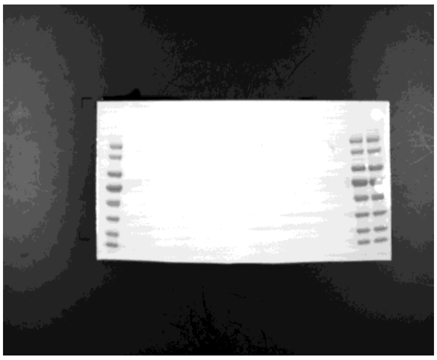

3rd

FAP	α-SMA
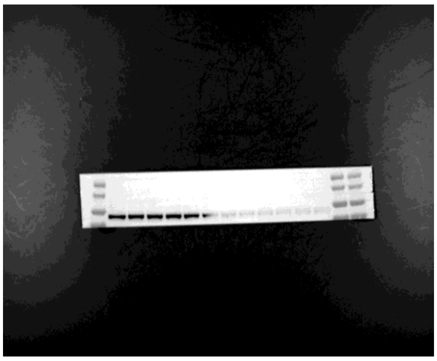	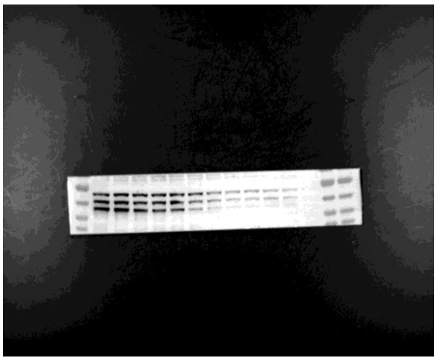
actin	white
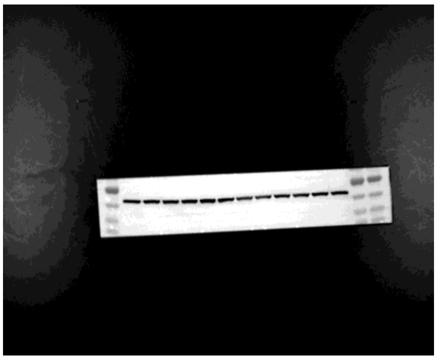	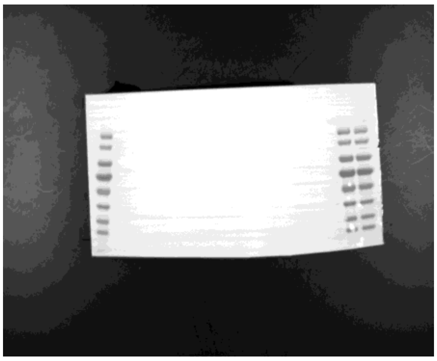

Fig. 4E

1st

FAP	α-SMA
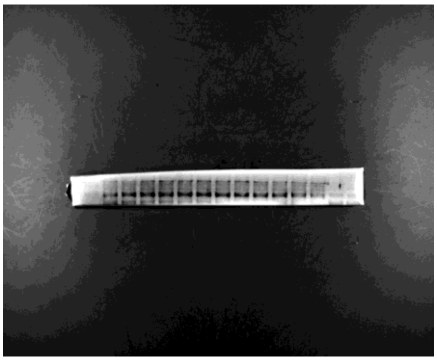	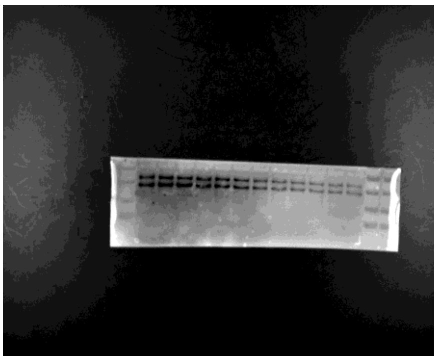
actin	white
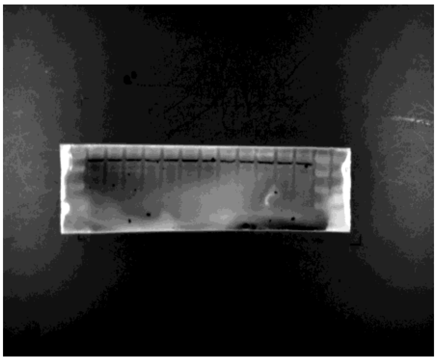	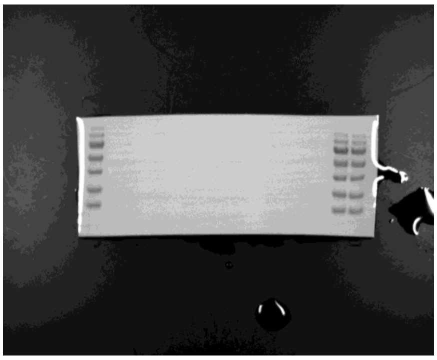

2nd

FAP	α-SMA
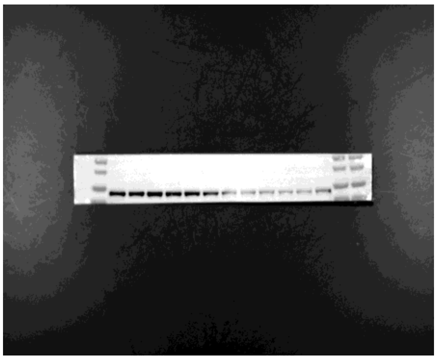	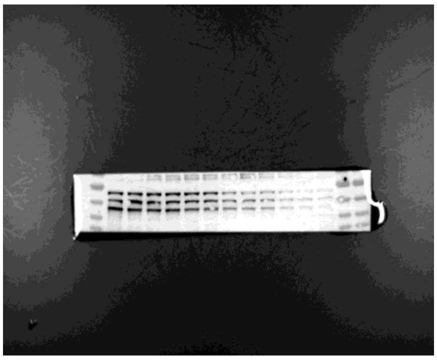
actin	white
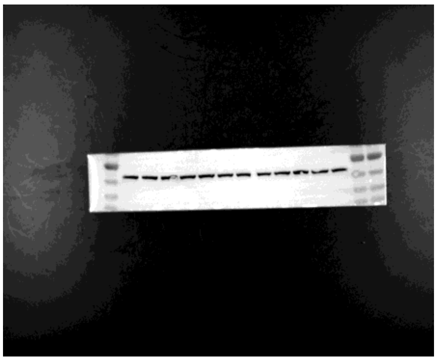	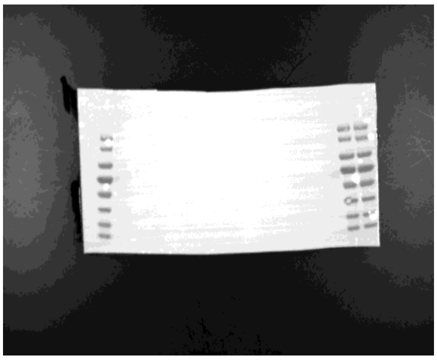

3rd

FAP	α-SMA
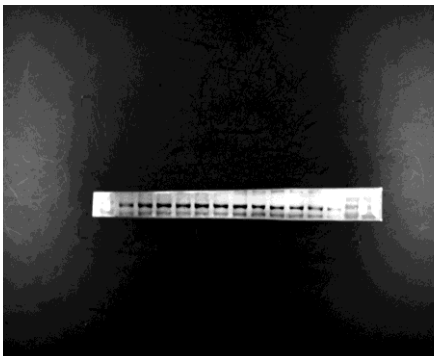	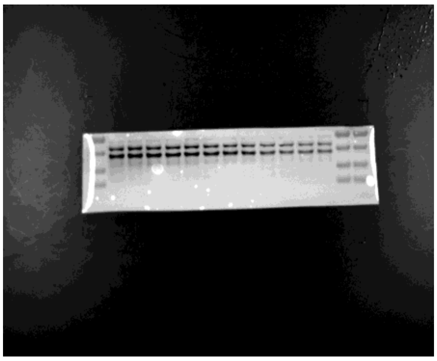
actin	white
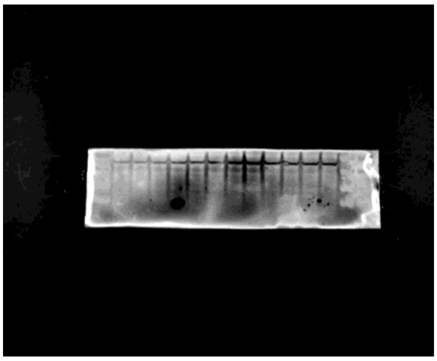	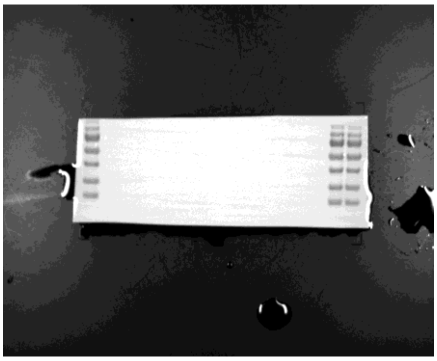
